# The Impact of a Small Private Online Course as a New Approach to Teaching Oncology: Development and Evaluation

**DOI:** 10.2196/mededu.9185

**Published:** 2018-03-05

**Authors:** Charlotte Vaysse, Elodie Chantalat, Odile Beyne-Rauzy, Louise Morineau, Fabien Despas, Jean-Marc Bachaud, Nathalie Caunes, Muriel Poublanc, Elie Serrano, Roland Bugat, Marie-Eve Rougé Bugat, Anne-Laure Fize

**Affiliations:** ^1^ Surgery Oncology Department Centre Hospitalier Universitaire de Toulouse, Institut Universitaire du Cancer de Toulouse-Oncopole Toulouse France; ^2^ Faculté de Médecine Université Paul Sabatier Toulouse III Toulouse France; ^3^ Department of Internal Medicine Centre Hospitalier Universitaire de Toulouse, Institut Universitaire du Cancer de Toulouse-Oncopole Toulouse France; ^4^ Pôle Hospitalo-Universitaire en Cancérologie, Cancer Pharmacology of Toulouse-Oncopole and Region (Work Package 4 Research Program) Institut Universitaire du Cancer de Toulouse-Oncopole Université Paul Sabatier Toulouse III Toulouse France; ^5^ Medical and Clinical Pharmacology Unit Centre Hospitalier Universitaire de Toulouse Toulouse France; ^6^ Radiotherapy Department Institut Claudius Regaud, Institut Universitaire du Cancer de Toulouse-Oncopole Toulouse France; ^7^ Support Care Department Institut Claudius Regaud, Institut Universitaire du Cancer de Toulouse-Oncopole Toulouse France; ^8^ Clinical Research Department Institut Claudius Regaud, Institut Universitaire du Cancer de Toulouse-Oncopole Toulouse France; ^9^ General Practice Department Université Paul Sabatier Toulouse III Toulouse France

**Keywords:** oncology, health education, continuing education, e-learning, SPOC, small private online course, education, medical, education, medical, continuing

## Abstract

**Background:**

Oncology involves complex care and multidisciplinary management of patients; however, misinformation and ineffective communication remain problematic.

**Objective:**

The educational objective of our study was to develop a new teaching method to improve cancer treatment and management by emphasizing the link between hospitals (inpatients) and their surrounding communities (outpatients).

**Methods:**

A team of 22 professionals from public and private institutions developed a small private online course (SPOC). Each offering of the course lasted 6 weeks and covered 6 topics: individual health care plans, cancer surgery, ionizing radiation, cancer medicines, clinical research, and oncological supportive care. For participants in the course, we targeted people working in the cancer field. The SPOC used an active teaching method with collaborative and multidisciplinary learning. A final examination was offered in each session. We evaluated participants’ satisfaction rate through a questionnaire and the success of the SPOC by participants’ completion, success, and commitment rates.

**Results:**

Of the total participants (N=1574), 446 completed the evaluation form. Most participants were aged 31 to 45 years. Participants included 56 nurses, 131 pharmacists, 80 from the medical field (including 26 physicians), 53 from patients’ associations, 28 health teachers, and 13 students (medical and paramedical). Among the participants, 24.7% (90/446) had an independent medical practice, 38.5% (140/446) worked in a public institution, and 36.8% (134/446) worked in a private institution. After completing the SPOC sessions, 85.9% (384/446) thought they had learned new information, 90.8% (405/446) felt their expectations were met, and 90.4% (403/446) considered that the information had a positive impact on their professional practice. The completion rate was 35.51% (559/1574), the success rate was 71.47% (1025/1574), and the commitment rate was 64.67% (1018/1574). Concerning the cost effectiveness of SPOC compared with a traditional classroom of 25 students, online education became more effective when there were more than 950 participants.

**Conclusions:**

SPOCs improved the management of oncology patients. This new digital learning technique is an attractive concept to integrate into teaching practice. It offered optimal propagation of information and met the students’ expectations.

## Introduction

International business schools and large companies have evolved in their thinking about new forms of teaching and collaborations, whereas medical universities retain a classical teaching approach [1]. Over the last few years, many massive open online courses (MOOCs) and small private online courses (SPOCs) have taught millions of students in virtual classrooms, changed learning techniques, and redefined the traditional boundaries in university teaching [2-6]. This digital learning is a new and attractive concept to integrate into teaching methods. One major positive benefit is its wide accessibility (“anytime, anywhere, on any device”). However, a significant problem with MOOCs is their completion rate of less than 10%, with an additional drop-off rate within the first week of the course [7].

In oncology, we constantly seek new approaches to improve the management of patients. The use of MOOCs seems to be supported by some parts of the oncological community, as demonstrated in the MOOC *Diagnostic Strategies of Cancers*, which opened in autumn 2016. The first results seem promising, with 23% of participants being successfully certified [4,5].

We are also supported by improvements in communication and networking between hospitals and different caregivers, including general practitioners, nurses, physiotherapists, pharmacists, and the medical community at large. Indeed, the complexity of medical care (especially in oncology), with the multidisciplinary management of patients, is not optimal due to some ineffective communication and misinformation [8-12]. To share information and experiences, practitioners could create an environment in which individuals can express concerns and alert team members to unsafe situations.

Thus, we decided to create a SPOC, which is limited to an invited audience, whereas a MOOC is generally open to all. The educational objective was to develop a new teaching method that could help to improve cancer treatment and its management by emphasizing the community–hospital interface.

To evaluate the relevance of this new teaching method, we analyzed the main characteristics of participants, their satisfaction with the course through a feedback questionnaire, and the cost effectiveness of the SPOC compared with theoretical face-to-face education.

## Methods

Our objective was to form a strong link between all participants on the same topic. To reach our objective, we used the following methods: (1) a SPOC with free user access, (2) oncology-specific information delivered by specialists, (3) a virtual platform that allowed for discussion and meetings on oncological matters, and (4) a final evaluation.

### SPOC Development Team

The development team comprised 22 professionals specializing in cancer care plus French faculty members. We divided the team into 2 subgroups: a teaching group and a project management group. Among the 22 professionals, 12 worked in a hospital, 8 worked outside the hospital (in the community), 1 specialized in the hospital–community network (and worked in the hospital and the community), 1 was an industrial pharmacist, and 1 worked within an institution (ie, Director of a Regional Health Agency).

The teaching team for the course was composed of 8 coordinator lecturer surgeons, radiotherapists, internal medical practitioners, clinical pharmacologists, a clinical researcher, and supportive care specialists; 14 other lecturers (senior nurses, hospital pharmacists, and physicians); and 8 general practitioners, dispensary pharmacists, physiotherapists, nurses, dentists, research and development managers from the pharmaceutical industry, patients, and caregivers, who provided testimonies. Teachers were volunteers and were chosen by the teaching council (ie, the course manager that introduced the SPOC, the project manager, and the Dean of the Medical Faculty of Toulouse III University). Among the 8 coordinators, we chose 6 lecturers for the 6 weeks of the SPOC, as they were specialists at our hospital (Institut Universitaire du Cancer de Toulouse-Oncopole, Toulouse, France). These 6 coordinators then chose 10 other assistant lecturers to help explore the specialties.

The teaching team and the SPOC project were managed by the project management team, which comprised 4 professionals: a project manager to oversee the project, a social officer to manage registration and forum moderation, a communications officer in charge of advertising, and a technical officer responsible for managing the multimedia elements.

### Target Learners and Accessibility

Participation in the course was open to all people involved in the management of cancer patients. Our public population included health students (medical and paramedical), health professionals, and members of patient associations.

Two sessions of the SPOC have been available since October 2016, but the offerings were not linked. Registration in the first session was not needed to register in the second session.

We initially offered open access to the SPOC to facilitate dissemination of knowledge, but then decided to restrict registration to students and professionals, and then, only after verifying their motivation, we also included volunteers from patients’ associations.

We limited the first offering of the course to 600 participants, whereas we did not limit inclusion for the second offering. The first SPOC opened its virtual doors on October 24, 2016, and the second on March 27, 2017. Participants registered on an outside platform and provided their characteristics. The registration home page also asked for the student’s identification number or health professional identification number [13].

### Course Content

We built the SPOC according to previously published information [14,15]. We planned 2 offerings of the course, each lasting 6 weeks, plus 3 supplementary weeks to provide adequate time for students to prepare for the final examination or to finish chatting on the forum.

**Figure 1:**
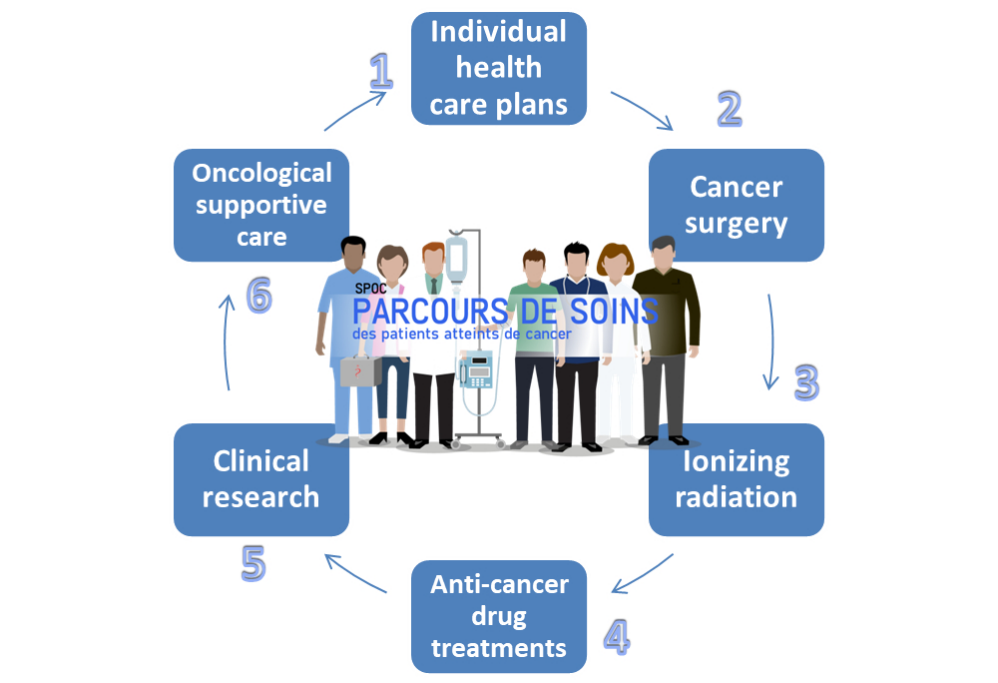
The 6 topics of the 6-week small private online course covering the sequential steps in the care of cancer patients.

The course covered 6 topics—week 1: individual health care plans; week 2: cancer surgery; week 3: ionizing radiation; week 4: cancer medicines; week 5: clinical research; and week 6: oncological supportive care (Figure 1).

These 6 topics were chosen by the teaching council. For each topic, at least 5 subthemes were determined in collaboration with the coordinator lecturer, and each subtheme was the subject of a video, as detailed in Table 1.

Each week, depending on the video that was shown, between 1 and 3 quizzes were presented (with a total of 75 quizzes for the 6 weeks). These consisted of multiple-choice questions with answers of true or false, fill-in-the-gap exercises, and drag-and-drop exercises.

The training was personalized: each participant could learn step-by-step and could choose his or her own learning pace. They could stop or rewatch videos, which was especially pertinent for difficult topics, or students could accelerate through a video if they were familiar with the subject. After the first session, we modified interactive parts of the forum and some of the exercises for the second session.

### Social Interactive Platform

To create social and active online communication, we opened a forum and links to Facebook, Twitter, and LinkedIn for discussion. We had 23 groups of 10 or 11 participants each who worked on 3 subjects: the new secondary effects of a drug, clinical trials, and supportive care. A webinar was suggested after 3 weeks (in the middle of the SPOC). Participants could then direct questions directly to a professional in the field.

**Table 1:** Topics covered in the small private online course and details of the accompanying videos.

Topic	Subject of video
Introduction	Cancer as a chronic disease
Week 1: Individual health care plans	Development of personalized care programs What are the elements of the health care pathway? A closer look at the multidisciplinary team meeting Link between health care facilities and home Care coordination mechanisms Interviews: Overview of the health care pathway and how to be helpful and patient—first-hand account
Week 2: Cancer surgery	Role of surgery in cancer treatment Various types of surgery Making the surgery less invasive Multidisciplinary preoperative planning is vital Postoperative follow-up Interviews: Role of the physical therapist in private practice
Week 3: Ionizing radiation	What is radiation therapy? Use of external radiation therapy Side effects of radiation therapy Innovations in radiation therapy What is brachytherapy? Different types of brachytherapy Interviews: Role of the dental surgeon
Week 4: Cancer medicines	Overview of drug-based (chemotherapy) treatment strategies The medication circuit (full motion design) Innovative cancer drugs Side effects Pharmacovigilance and adverse drug reaction reporting Individualized patient monitoring Initiation of oral cancer chemotherapy: important messages Interviews: Perspectives of a drug manufacturer, focus on reconciling drug treatments, role of the retail pharmacist
Week 5: Clinical research	Clinical research: general principles Joining a clinical trial Conduct of a clinical trial Monitoring of a patient participating in a clinical trial Focus on chemotherapy assistance by phone (EVAL COACH) Focus on a multidisciplinary meeting on going back home (CREDO) Interviews: Role of the primary care physician
Week 6: Oncological supportive care	What is supportive cancer care (SCC)? Various types of SCC (Part I) Various types of SCC (Part II) Fundamental role of patient education programs A continuously evolving field Interviews: Role of visiting nurses and home health aids
Conclusion	Cancer affects everyone

### Evaluation

Throughout the SPOC, we evaluated each participant’s satisfaction through a questionnaire that included 29 questions, which were validated by the teaching and communication teams. Each question could be answered using a 4-point scale. Categorical data were presented using numbers and percentages. Participants completed an evaluation form so that we could assess their characteristics and their expectations before starting the SPOC. Participants could also complete a satisfaction feedback form after the course. The SPOCs were certified for continuing professional development (CPD) by the Développement Professionnel Continu, according to French national recommendations (Haute Autorité de Santé). A final examination was suggested for each course. Each participant obtained a continuing health training certificate if their final score was greater than 50% (or 10/20), as recommended for CPD programs. The feedback form was designed by the project management team according to the French national recommendations for CPD programs.

We evaluated the success of the SPOCs according to the completion rate (percentage of participants who completed the 6-week course out of the total number of participants registered), success rate (percentage of participants who successfully completed the examination), and commitment rate (percentage of participants who completed the 6-week course out of the total number of participants who completed the first week of the course). We performed descriptive analyses on our population. All qualitative variables are described by numbers and percentages. Categorical variables are expressed as counts and frequencies (percentages). Quantitative variables, following a Gaussian distribution in our study, are described by their means and standard deviations. We used the chi-square test to compare results between the 2 SPOC offerings.

### Ethical Considerations

Our SPOC sessions were hosted by a private Web platform that respected the ethical considerations for personal data, in agreement with French law. The details are available on the FunCampus [16] and 360 Learning websites [17].

Students provided information outside these platforms (eg, through our anonymous surveys). Since we did not consider this to be personal data, in agreement with French law (Commission Nationale de l’Informatique et des Libertés), we did not need any ethical approval [18].

### Cost Effectiveness

To compare the overall cost of the SPOC and the theoretical costs of comparable face-to-face education, we calculated the overall cost of the online course compared with traditional classroom teaching for the same number of participants (eg, 1000 participants). We assumed a group of 25 participants in a traditional classroom. We calculated the point where online education became more effective.

The overall cost included salaries of a project manager, course director, teachers, a community manager, a beta tester, legal assistance, a communication department, and an administrative partner (a private enterprise, specific to a SPOC).

## Results

### Access and Participants’ Characteristics in the First Session

The first course session began on October 24 and finished on December 31, 2016. It lasted 6 weeks plus 3 supplementary weeks to allow adequate time for students to prepare for the final examination.

Among the 600 participants, 176 completed the questionnaire, which provided the following data. Most participants were aged 31 to 45 years (range 18 to >50 years); 68.4% (120/176) of participants were aged over 46 years. The paramedical group was well represented, with 26.1% (46/176) being nurses. A total of 24 (13.8%) were pharmacists, pharmacy students, and dispensary pharmacists, and 17 (9.7%) were in the medical field (doctors, medical students, and residents). The completion rate of this SPOC was 36.0% (216/660), the success rate was 66.0% (396/600), and the commitment rate was 72.0% (432/600).

### Participants’ Characteristics and Course Modifications for the Second Session

The second session ran between March 27 and May 31, 2017. The course ran for 6 weeks plus 3 supplementary weeks to enable adequate time for students to prepare for the final examination.

We modified some elements of the SPOC for the second session, as follows. (1) Responding to the feedback form completed in the first SPOC, we rewrote some sentences and modified the format of the collaborative exercises. (2) To ensure that the second SPOC suited the participants, we created a beta test group from the first SPOC to test the format of the exercises and to detect any technical problems before starting the second SPOC. (3) A total of 6 volunteers from the first SPOC participated in moderating the forum, creating collaborative exercises, and encouraging engagement of the participants in the collaborative exercises. (4) We modified the format of the collaborative exercises. The initial format was a pluriprofessional discussion on one predefined subject, but there were few interactions. Each participant answered the question without considering the comments from the other participants. Thus, we switched to an exercise that fostered more collaborative homework. Moreover, participants had to evaluate the homework of the other groups.

In this second course session, of the 975 participants, 270 completed the questionnaire, which supplied the following data. Most participants were aged 31 to 45 years. The age distribution was the same as in the first course. Of the paramedical group, 42.9% (116/270) were nurses. A total of 124 (45.9%) were pharmacists, pharmacy students, and dispensary pharmacists, and 10 (3.7%) were medical students. The completion rate was 34.8% (339/975) the success rate was 76.9% (550/975) and the commitment rate was 56.9% (555/975).

### Evaluation of the 2 Sessions

Of the total 1574 participants, 446 completed the evaluation questionnaire. Most participants were aged 31 to 45 years. There were 121 paramedical and social workers (including 56 nurses), 131 pharmacists, 80 participants from the medical field (including 26 physicians), 53 participants from patients’ associations, 28 teachers, 17 administrative or industrial, 13 students, and 3 others (nonclassified) (Table 2).

**Figure 2:**
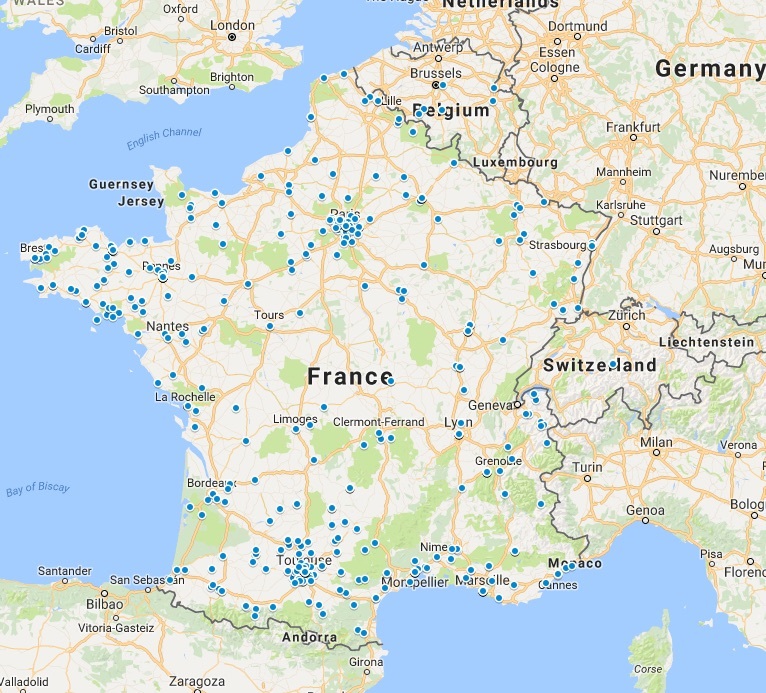
Geographic representation of participants from France. Each blue dot represents a connection to the small private online course.

**Figure 3:**
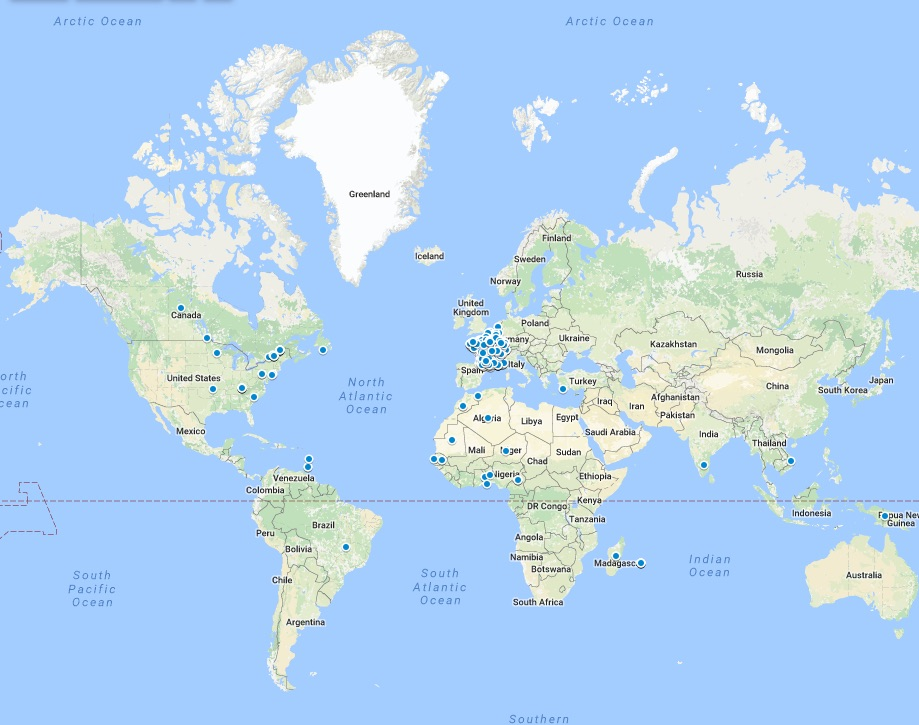
Geographic representation of participants from around the world. Each blue dot represents a connection to the small private online course.

Registration was open to participants from all countries as long as they could understand the French language; most participants lived in France (n=427, 95.7%) (Figure 2). Other participants mainly lived on African continent (n=6, 1.4%), the United States (n=6, 1.4%), or Canada (n=4, 0.9%) (Figure 3).

Among the participants, 24.9% (111/446) had an independent medical practice, 38.9% (173/446) worked in a public institution, and 36.9% (165/446) worked in a private institution.

**Table 2:** Characteristics of participants who completed the questionnaires in the first and second offerings of the small private online course.

Characteristic		First session (n=176)	Second session (n=270)	Overall results (n=446)
**Age range (years), n (%)**				
	18-25	40 (22.7)	21 (7.8)	61 (13.7)
	26-30	21 (11.9)	39 (14.4)	60 (13.5)
	31-45	59 (33.5)	125 (46.3)	184 (41.3)
	46-50	24 (13.6)	30 (11.1)	54 (12.1)
	>50	32 (18.2)	55 (20.4)	87 (19.5)
**Profession or specialty, n (%)**				
	Medicine	65 (36.9)	15 (5.6)	80 (17.9)
	Pharmacist	8 (4.5)	123 (45.5)	131 (29.4)
	Paramedical and social^a^	32 (18.2)	89 (33.0)	121 (27.1)
	Teachers	9 (5.1)	19 (7.0)	28 (6.3)
	Students	3 (1.7	10 (3.7)	13 (2.9)
	Administrative/industrial	7 (4.0)	10 (3.7)	17 (3.8)
	Patients’ association	50 (28.4)	3 (1.1)	53 (11.9)
	Other	2 (1.1)	1 (0.4)	3 (0.7)
**Place of practice**				
	Independent medical practice	N/A^b^	45 (24.7)	45 (24.7)
	Public institution	N/A	70 (38.5)	70 (38.5)
	Private institution	N/A	67 (36.8)	67 (36.8)

^a^Including nurses, physiotherapist and osteopath, psychologists, social workers, nurse’s aide, radiotherapist and radiologist technician, socioaesthetician, medical assistant.

^b^N/A: the question was not in the questionnaire during the first session.

**Figure 4:**
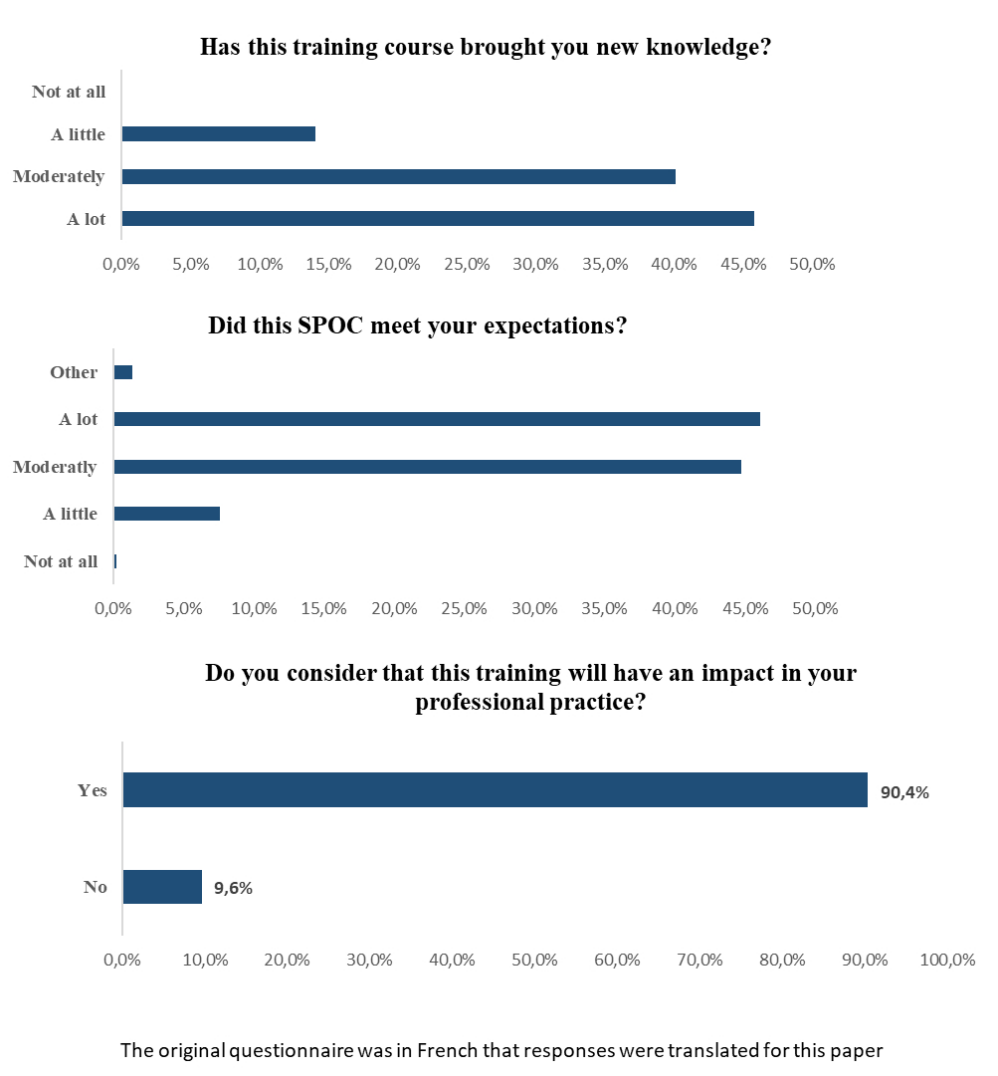
Representative responses to questions assessing satisfaction with the 2 sessions of the small private online course (SPOC). The original questionnaire was in French but responses were translated for this paper.

Among the participants, 85.9% (383/446) thought they had learned new information (moderately: 179/446, 40.2%; considerably: 205/446, 45.9%), 90.8% (405/446) felt their expectations had been met (fully: 204/446, 45.7%; relatively: 201/446, 45.1%), and 89.9% (401/446) considered the course had a positive impact on their professional practice as a caregiver (Figure 4). The completion rate was 36.02% (567/1574), the success rate was 71.98% (1133/1574), and the commitment rate was 64.99% (1023/1574).

### Interactive Social Platform

Many participants expressed their satisfaction (or dissatisfaction) by posting comments on the forum during the courses. In total, 2812 comments were posted on videos and the forum: 2367 “liked” (the first session) and nearly 84.9% (379/446) considered it positive, with 15% suggesting improvements.

### Cost Effectiveness

**Figure 5:**
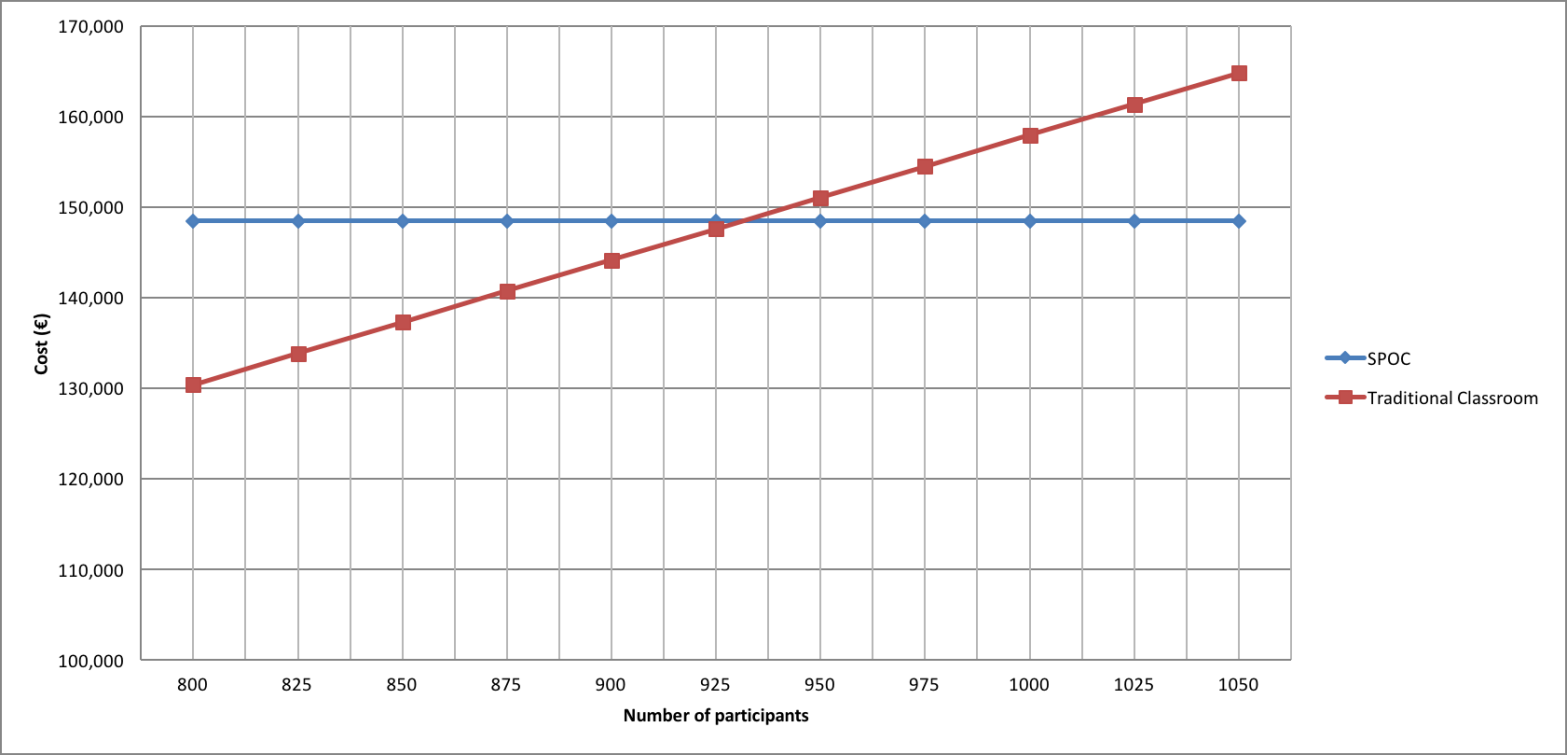
Cost comparison between the small private online course (SPOC) and traditional face-to-face classroom education.

There was an initial and fixed high cost to developing the SPOC, independently of the number of participants. The only costs that could be modified were the animations, the forum, and the registration platform. In contrast, traditional face-to-face classrooms have a low cost initially, but this then increases according to the number of students. For example, for 1000 participants, the overall cost of the SPOC was €148,000, versus €154,000 for face-to-face education (€154 per student). If we assumed a classroom with 25 students, the point where online education became more effective was 950 participants (Figure 5).

## Discussion

### Principal Findings

This SPOC was a positive experience with a completion rate of 35.5%, a success rate of 71.5%, and a commitment rate of 64.7%. Among the 1574 participants, most were aged 31 to 45 years and were paramedical practitioners (nurses, pharmacists). Most (90.4%) considered that this course had a positive impact on their professional practice. The cost effectiveness of this online education became more effective at more than 950 participants.

This SPOC was an innovative oncological teaching method and had a high completion rate (35.5%). In general, on MOOC platforms, the completion rates are between 5% and 10% [7]. Hoy suggested that, in medicine, MOOCs could be used in continuing medical education [19]. However, few medical universities use this kind of teaching [4,5,15,20-22].

A high completion rate (38%) was observed in an Australian MOOC on dementia: 4409 registrants took part in discussion boards and 3624 completed the course [23]. In addition, a French MOOC that opened in 2016, the *Diagnostic Strategies of Cancers*, had a satisfactory completion rate (23%) [4,5].

From that experience, we decided to design a SPOC that was a modified MOOC. In our SPOC, the number of registered participants and the completion rate in the second session (341/975, 34.9%) were similar to rates in the first session (261/600, 36.0%). This demonstrated that students’ interest in this SPOC remained high and was not only an initial enthusiasm for a new teaching method. Also, the large difference in completion rates between MOOCs and our SPOC could be because SPOCs are developed for a targeted audience and are, therefore, able to better suit the educational needs and interests of their participants [3-5,7].

In the MOOC *Diagnostic Strategies of Cancers*, there were 2 types of learners: students in health and biology, and members of the general public. Of the participants, 71% chose to go on to student teaching. Of the 5285 participants from 81 different countries, 1237 (23%) were successfully certified [4,5].

In our SPOC, we targeted the participant profile at medical and paramedical caregivers to create a stronger link between professionals sharing an interest in oncology. We found that nurses were the main group represented in the first session, whereas pharmacists were the main group in the second session. This could be because more information was delivered by the pharmaceutical community about this SPOC after the first course offering. Moreover, regarding age, we found that most participants were not very young: 41% were aged 31 to 45 years, 20% were aged over 50 years, and 12% were aged 46 to 50 years. Yet digital learning is a modern methodology.

The SPOC allowed experiences to be shared. The participant did not need to train alone in front of his or her computer but could also be involved in social and collaborative work. In fact, Uijl et al [24] recently evaluated 4 courses from the University Medical Center Utrecht’s international Master’s Program in Epidemiology. The 71 included students benefited from extended social interactions during the SPOC. There were around 1500 interactive posts across the 4 courses, in 575 discussions, of which 43% were social discussions. Of these, 90% were initiated by students, and 94% was aimed at students. The authors of this study concluded that the SPOC had a sustainable concept and created an environment suitable for learning, thus fitting with the need for social interactions in higher education [24]. The same results have been observed in other studies [25-27], as well as in our SPOC.

In the second course offering, we improved interactions between the participants through a dedicated forum with collaborative exercises. We deliberately mixed professionals from different areas and institutions: about one-third were in private practice, one-third worked in a public hospital, and one-third worked in a private institution. The result of these interactions was further complemented by peer evaluation. All these activities contributed to the development of a social learning environment and (in large part) to the high completion rate. Indeed, most participants felt their expectations had been met (91%).

As shown in an online accreditation course, a professional practice forum improved learning outcomes through sharing expertise [28]. The SPOC seemed to be a good way to strengthen coherence and communication between the different caregivers (nurses, doctors, pharmacists, physiotherapists, etc). It became clear that digital learning improved communication and united the participants’ efforts on one subject, thus forming a link between those working in the hospital and those working in the surrounding community. In study of a MOOC on diabetes, Wewer Albrechtsen et al reported that, among 845 caregivers, the MOOC had a positive effect on their practice of 88% and extended the professional network of 48% [29].

One major positive aspect of this teaching method is its wide accessibility. As demonstrated in our SPOC, training was accessible from a participant’s computer or tablet from anywhere and at any time. We observed that 4.26% (19/446) of our participants lived outside France: they were either expatriates or foreigners who spoke French.

We analyzed the cost effectiveness of this type of education. In our study, we compared the overall cost of our SPOC with that of a traditional classroom for the same number of participants. We found that the online course was more cost effective when there were more than 950 participants. This may be for three main reasons. First, the overall cost of the SPOC was higher (because we chose a private enterprise as the service provider, outside of the university). Second, this SPOC was free of charge to participants, whereas many online teaching courses charge fees. Third, face-to-face education does not allow for the course to be offered in foreign countries (due to, for example, extra travel costs). Thus, the cost for SPOCs could be less than we have calculated. Other researchers have analyzed costs and compared them with costs of traditional education. In a randomized study, Nilsson et al [30] compared the cost effectiveness of a mobile app-guided versus a textbook-guided ultrasound course. Of the 34 participants who completed the course, there were no statistically significant differences in test performance or diagnostic accuracy between the 2 groups. Yet textbook-guided training was significantly more cost effective than mobile app-guided training [30]. Erbe et al described different methods to study common mental health disorders; they reported that blended interventions (combining the strengths of face-to-face and Internet approaches) were feasible and could be more effective [31]. More randomized clinical trials on the effectiveness and cost effectiveness of blended treatments are necessary. Recently, Tolsgaard and Cook discussed the costs and outcomes of improving educational programs according to their context. They concluded that, even if the costs and outcomes were individually very important, perceived value also must be considered in order to decide change of current educational practices [32].

Moreover, our questionnaire raised some points that need improvement. For example, participants commented on the general nature of the topics covered during the courses and would have liked more detail, for example, about the secondary effects of chemotherapy and radiotherapy. This feedback helped us improve the content and structure of the second SPOC offering. Indeed, SPOCs are constantly evolving, with the possibility that new modules can be added, and thus forums can be developed for discussion, collaborative exercises, and interactivity between participants. Our SPOC will be integrated further for medical and paramedical (nurse) educational institutions and universities in France. To evaluate the educational effectiveness of our SPOC, we plan to test retention of knowledge over time by sending an evaluation form to participants at 1 year after they have completed the SPOC.

### Conclusion

The clarity (information, support, access, registration, and content), communication (exercises and forum), and interactivity (assessment, collaborative exercises, and feedback) in our SPOC made this a good educational method for CPD and interprofessional education. This relatively new digital learning tool is an attractive concept to integrate teaching, especially in oncology. It offers optimal propagation of information in a cost-effective way and meets the students’ expectations for training.

## Abbreviations

CPD: continuing professional development
MOOC: massive open online course
SPOC: small private online course
